# Metabolic profiling and scavenging activities of developing circumscissile fruit of psyllium (*Plantago ovata* Forssk.) reveal variation in primary and secondary metabolites

**DOI:** 10.1186/s12870-020-2318-5

**Published:** 2020-03-14

**Authors:** Manish Kumar Patel, Avinash Mishra, Santlal Jaiswar, Bhavanath Jha

**Affiliations:** 1 Division of Applied Phycology and Biotechnology, CSIR- Central Salt and Marine Chemicals Research Institute, G. B. Marg, Bhavnagar, Gujarat 364002 India; 2 Present address: Department of Postharvest Science, Institute of Postharvest and Food Sciences, Volcani Center, Agriculture Research Organization, HaMaccabim Road 68, 7505101 Rishon LeZion, Israel

**Keywords:** Isabgul, Metabolite profiling, Plantago, Psyllium, Scavenging activity

## Abstract

**Background:**

Developing fruit is considered as an excellent model to study the complex network of metabolites which are altered rapidly during development.

**Results:**

Metabolomics revealed that developing psyllium fruit is a rich source of primary metabolites (ω-3 and ω-6 fatty acids and amino-acids), secondary metabolites and natural antioxidants. Eidonomy and anatomy confirmed that psyllium fruit followed five stages of development. Total lipids and fatty acids were synthesized differentially; saturated fatty acids (FAs) increased, whereas total polyunsaturated FAs decreased with increasing developmental stage. The unsaturation index and degree of unsaturation showed a catenary curve. Principal component analysis confirmed a significant shift in the FA profile from bud initiation to the maturation stage. Similarly, a similar level of total amino acids was present at different developmental stage following a temporal biosynthesis pathway. Total phenolic and flavonoid contents decreased in tandem with fruit development. Twenty-two different metabolites were identified, and metabolic changes were also observed during fruit development. Six metabolites were detected exclusively in the flowering stage, whereas two were detected in each of early and maturity stages of development. The metabolites apigenin and kaempferol were detected ubiquitously in all developmental stages. Time-dependent metabolomics revealed a shift in metabolite biosynthesis.

**Conclusion:**

During fruit development, metabolites, FAs, amino acids, total phenolics, total flavonoids, antioxidants and scavenging activities changed progressively and were co-ordinately linked to each other. As a future perspective, further studies will focus on the validation of identified metabolites, which integrated with transcriptomics data and will reveal the metabolic regulatory network of development psyllium fruit.

## Background

*Plantago ovata* Forssk. is an ancient annual herbaceous plant species of the Plantaginaceae family and widely cultivated in tropical regions of the world [8]. Psyllium spp. has been used since ancient times for the treatment of many health conditions and diseases, such as inflammation, wound healing and cancer [29]. “Isabgol husk” represents the epidermal layer of the seed, which is a pharmaceutically important part of the plant. Husk is a natural laxative, contains mucilaginous compounds of medicinal values and is used as a thickening agent in the pharmaceutical industry. It is traditionally used as a prophylactic agent in the treatment of bowel obstruction, constipation, diarrhoea and dysentery [8].

Psyllium, the common name of the *Plantago ovata*, contains phenolics, flavonoids, terpenoids and iridoid glycosides [54]. The fruiting body of psyllium possesses powerful antioxidant compounds and is considered a rich source of natural antioxidants [4, 54]. The flavonoids scutellarein 7-glucoside, scutellarein 7-glucuronide, scutellarein (5,6,7,4′-tetrahydroxyflavone), apigenin, luteolin, hispidulin (5,7,4′-trihydroxyS-methoxyflavone) and 5,7,4′,5′-tetrahydroxyflavanone-3′-O-glucoside are widely reported from psyllium species ([14, 17, 21, 28, 35]). Flavonoids are involved in mechanisms of defence against cardiovascular disease and in other in vitro antioxidant activities. More than 6000 varieties of flavonoids have been categorised, most of them reported from flowers, fruits and leaves of the plant [20]. Flavonoids interact with different types of enzymatic inhibitors, such as cyclooxygenase and lipoxygenase, which are involved in many biological activities, including antimicrobial, antihepatotoxic, antiosteoporotic, antiulcer, antiproliferative and apoptotic activities [1, 18]. For example, quercetin-3-O-α-L-rhamnopyranoside obtained from *Toona sinensis,* showed antioxidant and anticancer activities [65], kaempferol and 8-C-(1,1- dimethyl-2-propen-1-yl)-5,7-dihydroxyflavonol isolated from *Platanus orientalis* and *Platanus acerifolia* showed osteoblastogenic as well as ERα-mediated estrogenic activity [59], and kaempferol and hyperoside are the active compounds in seeds of *Cuscuta chinensis*, which have an osteogenic effect [62]. As a dietary or medicinal supplement, polyphenols and flavonoids contain health-promoting properties due to their antioxidant activities [10]. Heterogeneous compounds, such as polyphenols, are involved in antioxidant activities such as ABTS (2, 2′-azinobis-(3-ethylbenzothiazoline-6-sulfonic acid) scavenging, DPPH (2, 2-diphenyl-1-picrylhydrazyl) scavenging and hydroxyl radical scavenging [2].

Reactive oxygen species (ROS) are generated in response to stress, causing oxidative damage to the cell; therefore, plants exhibit robust defence mechanisms against ROS-induced oxidative stress [7, 25, 44, 60]. Several mechanisms are involved in free-radical scavenging activity, such as destruction of ROS formation by inhibition of enzymes that are involved in free-radical generation, ROS scavenging and protection by antioxidant defences [7, 40]. The plant metabolomics approach has been used to identify and quantify primary and secondary metabolites of medicinal plants [33, 39, 45]. Plant primary metabolites, such as fatty acids, amino acids and carbohydrates, are essential to life, whereas the production of secondary metabolites is co-ordinated with respect to plant development [42, 43]. Plant secondary metabolite contents are influenced by genotype, phenological stage and ecophysiological conditions [31]. The phenological stage is considered as the most important determining factor of the quality and quantity of metabolites from dietary, nutritional and pharmaceutical points of view and thus holds great significance [31]. Previously, we reported on comparative metabolic profiling of different plant parts (e.g. leaves, seeds and husks) of psyllium [45]. In the previous report, the nutraceutical potential of this plant was revealed, and it was also suggested that psyllium leaves could be used in a green salad as a dietary supplement to daily food [45]. Polysaccharides obtained from psyllium leaves, seed and husk have potential to be used as natural antioxidant and also have anti-proliferative activity [42, 43]. Seed development is an essential process of psyllium. Flower and seed development is characterized by structural changes, such as floral initiation, floral organ formation, floral differentiation and growth, leading to seed maturity [5]. The fruiting bodies of psyllium are a potent reservoir of primary and secondary metabolites. The organoleptic and nutritional qualities of a fruit are dependent on the total metabolite composition, which varies throughout the developmental stages [34]. Studying the chemical composition of developmental stages associated with specific metabolism and free-radical scavenging activity would be of immense importance. However, to the best of our knowledge, no research has been done, so far, on the metabolite profiling, scavenging analysis and morphological study of the developing fruiting body of psyllium. Therefore, the present study was carried out, and metabolite profiling of the developing fruit was performed to determine the potential candidate or appropriate harvesting time for the fruiting body of psyllium for nutraceutical applications. In the present study, metabolomics and scavenging activities of developing fruit were analysed using modern analytical tools and techniques [46] including gas chromatography-mass spectrometry (GC-MS), high-performance liquid chromatography (HPLC) and liquid chromatography-mass spectrometry (LC-MS). The present study provides useful insight into the morphological, biochemical and metabolic responses of the developing fruiting body of psyllium. Furthermore, the present study will also provide guidance for the optimisation of the harvesting time of psyllium fruits and the maximisation of health benefits.

## Results

### Eidonomy and anatomy of developing circumscissile capsule

**Fig. 1:**
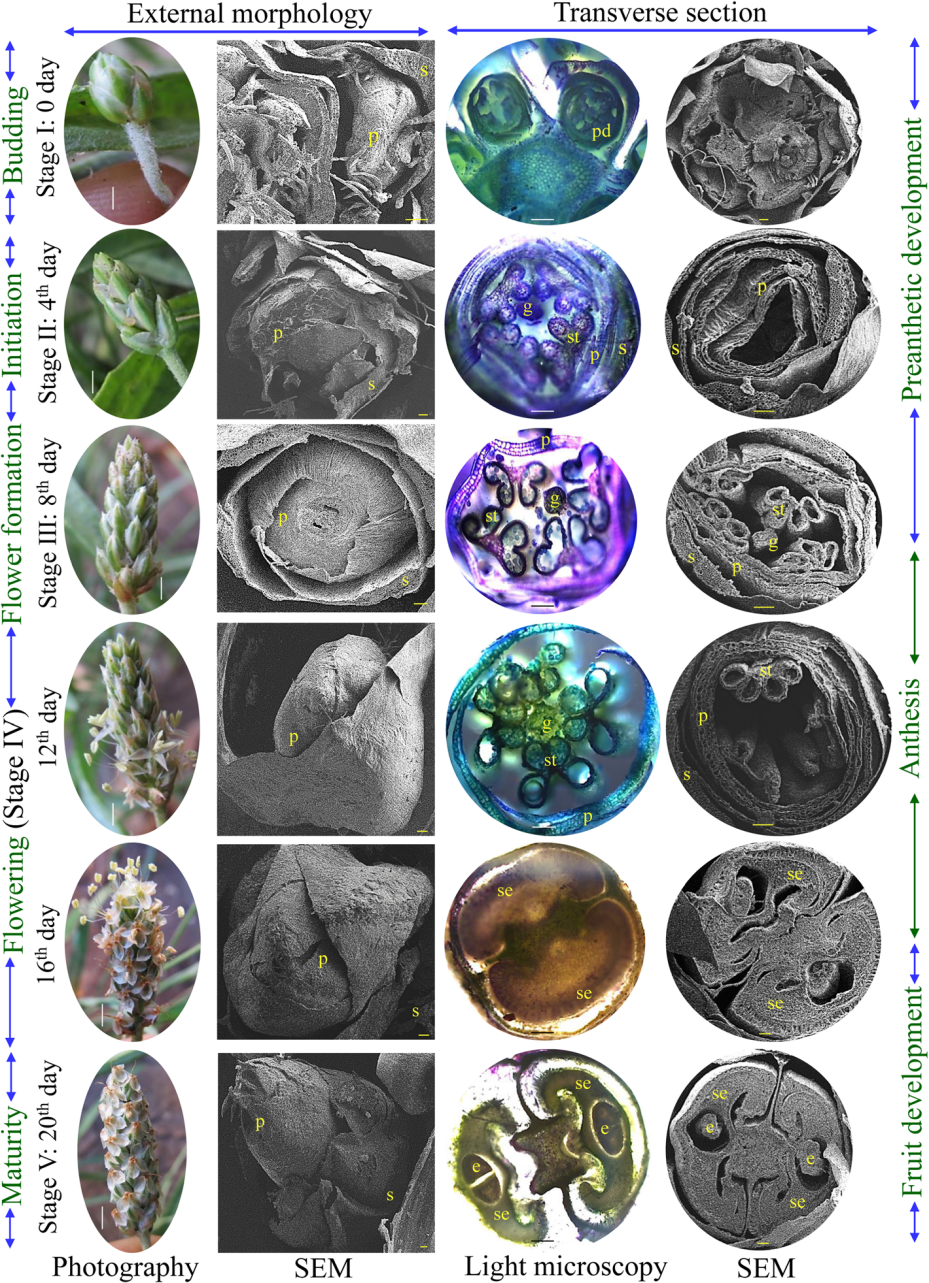
Flower development of psyllium (*Plantago ovata*). Eidonomy and anatomy studies were monitored from flower bud induction (day zero) to fruit maturity (day twenty) under photography and scanning electron microscopy. Abbreviations: e, embryo; g, gynoecium; p, petal; pd., presnthetic development; s, sepal; se, seed endosperm; st, stamens. Scale bar of first column (i.e. photography) shows 0.5 cm while others represent 0.1 mm

**Fig. 2:**
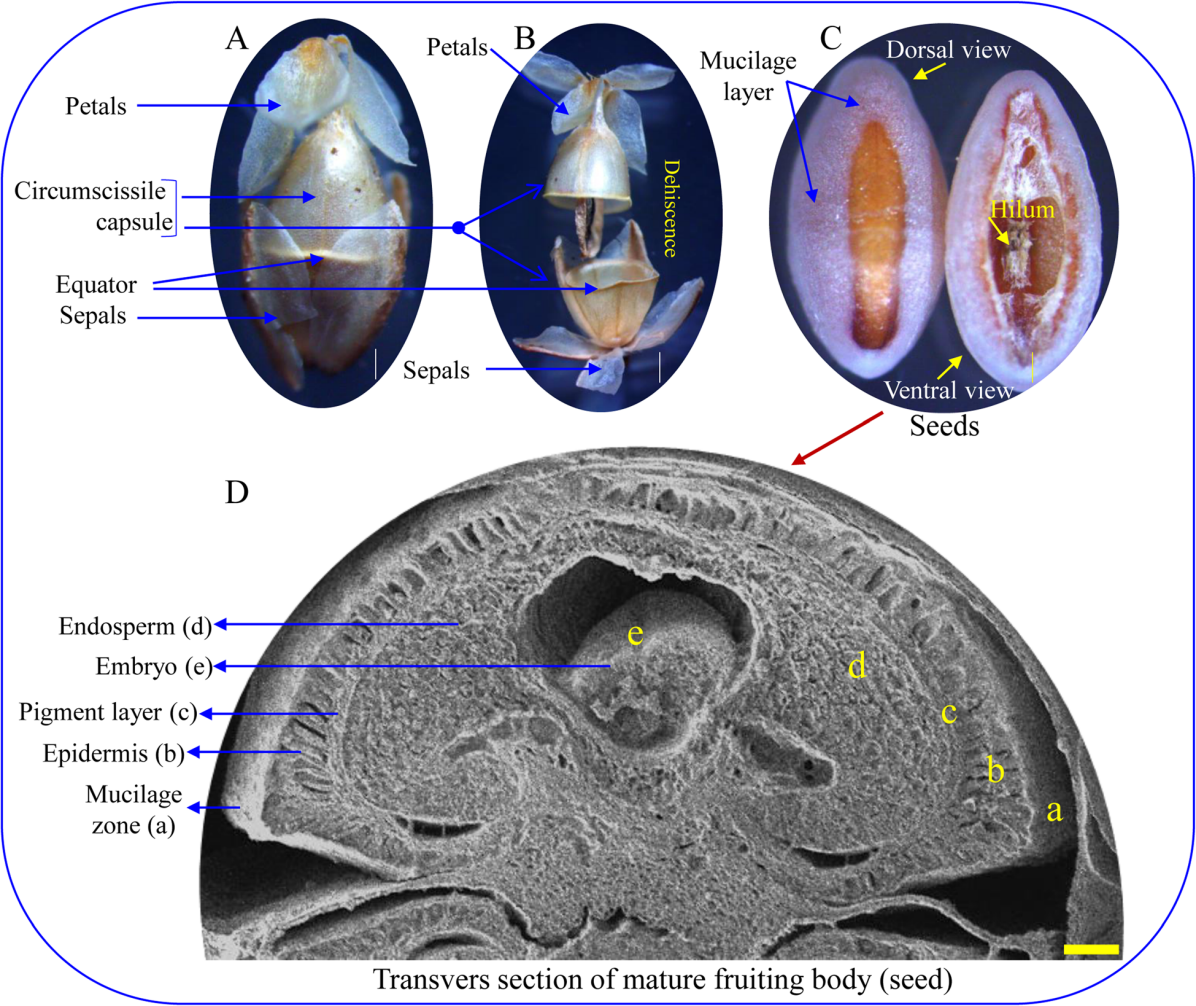
Analysis of circumscissile capsule (fruit) of psyllium. **a** Mature fruit, (**b**) Dehiscent fruit, (**c**) Seed morphology and (**d**) Transverse section of mature seed

The colour of the fruiting body changed gradually from green to red throughout the different stages of development. Commonly, psyllium fruits reach maturity within 20 days and follow different developmental stages (Fig. 1). A total of five stages were observed: floral budding (stage-I; 0 day), floral organ initiation (stage-II; 4th day), floral organ formation (stage-III; 8th day), full flowering (stage-IV; 12th to 16th days) and fruit maturity (stage-V; 20th day). Eidonomy observations revealed that the inflorescence primordium originated from the axile of the leaves and developed into the spike primordium at later stages. In psyllium, a number of inflorescences ascended from the base of the plant (Fig. 1). Flowers were numerous, small and white; arranged in four spiral rows on the ovoid or cylindrical spikes and consisted of four sepals, four petals, four stamens and a pistil. Sepals were free, concave, glabrous and elliptic, whereas petals were glabrous, reflexed and white. The psyllium ovary was free and bilocular (single ovule per locule) with axile placentation. Acropetalous types of inflorescences, in which older flowers grew towards the base while younger flowers were at the growing end, were observed in psyllium. At the early stage of inflorescence development (stage I, 0 day), the spike was 5 mm in length, and the transverse section showed an initial stage of five flower differentiations. Of these, three flowers showed developing sepals, petals and stamens, whereas two contained sepal and petal primordia. An initiation of anther with four stamens and gynoecium was detected at stage II (4th day), which was clearly seen in later stages of development (stage III, 8th day onwards). Seed development was initiated from stage IV (12th to 16th days), and fruit reached maturity on the 20th day of development (stage V). The mature fruit of psyllium is known as a circumscissile capsule, which is divided horizontally into two parts during dehiscence from the equator to release the seeds (Fig. 2). Psyllium seeds are translucent, hard, yellowish brown, smooth, boat-shaped and covered with a mucilage layer. The presence of a hilum was noticed on the concave (ventral) side of the seed, which was covered with a thin, white membrane (Fig. 2). Seeds did not emit any odour or have any taste, but the succulent part was bitter and astringent. The transverse section of a mature seed revealed differentiated endosperm, embryo, pigment layer and epidermis (Fig. 2).

### Fatty acids differentially synthesized in developing fruiting body

The total lipid and fatty-acid (FA) composition were identified and quantified at different developmental stages (0, 4th, 8th, 12th, 16th and 20th days) of psyllium fruit (Table 1). The maximum content of saturated FA (SFA) was detected at stage I (0 day; 49.7%) and declined up to stage III (8th day), at which was found the lowest content (25.7%), after which it increased, reaching up to 33.7% at maturity (stage V, 20th day). In contrast, the total polyunsaturated fatty acid (PUFA) increased with developmental stage and reached a maximum at the 8th day (stage III; 72.5%), thereafter decreasing with increasing developmental stage. Among PUFAs, C18 PUFAs were dominant (48–71% of total FAs) over C20, which were found in traces (about 0.5–2% of total FAs) in the developing fruiting bodies. The unsaturation index (UI) and degree of unsaturation (DU) showed a catenary curve with maximum values of 188.3 and 146.9, respectively at stage III (8th day).

**Table 1:** Total lipid and fatty acid composition of developing fruiting body of psyllium

Fatty acids	Name of fatty acids	0 day (Stage I)	4th day (Stage II)	8th day (Stage III)	12th day (Stage IV)	16th day (Stage IV)	20th day (Stage V)
*Lipid composition*							
∑ SFA		49.71 ± 1.46	30.12 ± 1.98	25.66 ± 0.45	28.43 ± 0.65	31.14 ± 1.25	33.65 ± 2.86
∑ MUFA		2.31 ± 0.72	1.96 ± 0.01	1.84 ± 0.01	1.73 ± 0.08	2.07 ± 0.13	3.16 ± 0.93
∑ PUFA		47.98 ± 0.74	67.92 ± 1.99	72.51 ± 0.44	69.84 ± 0.73	66.79 ± 1.38	63.18 ± 3.80
∑ 18 PUFA		47.61 ± 0.75	66.78 ± 2.20	70.61 ± 0.81	68.19 ± 0.85	65.4 ± 1.85	61.48 ± 4.96
∑ 20 PUFA		0.37 ± 0.01	1.14 ± 0.21	1.90 ± 0.37	1.64 ± 0.13	1.39 ± 0.46	1.70 ± 1.17
UI		119.25 ± 0.51	174.56 ± 3.91	188.25 ± 0.91	180.57 ± 0.09	171.52 ± 3.75	153.78 ± 8.15
DU		98.26 ± 2.21	137.8 ± 3.97	146.85 ± 0.88	141.41 ± 1.38	135.64 ± 2.63	129.53 ± 6.66
n6/n3		1.3 ± 0.24	1.10 ± 0.05	1.01 ± 0.01	1.00 ± 0.09	1.05 ± 0.06	2.10 ± 0.17
n9/n3		0.11 ± 0.04	0.06 ± 0.01	0.05 ± 0.01	0.04 ± 0.01	0.06 ± 0.01	0.14 ± 0.06
n9/n6		0.08 ± 0.02	0.06 ± 0.01	0.05 ± 0.01	0.04 ± 0.01	0.05 ± 0.01	0.06 ± 0.03
AI		31.29 ± 3.11	38.72 ± 0.39	39.72 ± 0.79	38.14 ± 0.54	38.86 ± 0.77	48.01 ± 0.93
TI		97.73 ± 4.25	120.52 ± 0.48	129.41 ± 0.67	128.12 ± 3.31	122.46 ± 2.25	87.19 ± 4.95
*Fatty acids composition*							
C12:0	Lauric acid	n.d	n.d	n.d	n.d	0.77 ± 0.59	0.63 ± 0.21
C14:0	Myristic acid	0.27 ± 0.01	0.17 ± 0.02	0.16 ± 0.02	0.24 ± 0.01	0.29 ± 0.01	n.d
C14:1	Myristoleic acid	n.d	n.d	n.d	n.d	n.d	0.14 ± 0.02
C15:0	Pentadecanoic acid	0.20 ± 0.01	0.18 ± 0.01	0.2 ± 0.02	0.23 ± 0.01	0.26 ± 0.01	0.22 ± 0.02
C16:0	Palmitic acid	32.53 ± 0.73	22.61 ± 0.39	20.23 ± 0.28	21.96 ± 0.11	23.68 ± 0.97	24.53 ± 0.46
C16:1 (n-7)	Palmitoleic acid	n.d	n.d	0.19 ± 0.01	0.18 ± 0.02	0.24 ± 0.01	0.27 ± 0.02
C17:0	Heptadecanoic acid	0.18 ± 0.01	0.16 ± 0.01	0.18 ± 0.01	0.20 ± 0.02	0.25 ± 0.01	0.23 ± 0.01
C17:1 (n-7)	Cis-10-Heptadecanoic acid	n.d	n.d	n.d	n.d	n.d	0.06 ± 0.02
C18:0	Stearic acid	15.31 ± 0.91	5.37 ± 1.62	3.41 ± 0.61	4.15 ± 0.55	4.44 ± 0.60	6.55 ± 2.48
C18:1 (n-9)	Oleic acid	2.14 ± 0.72	1.47 ± 0.07	1.41 ± 0.05	1.34 ± 0.10	1.65 ± 0.13	2.65 ± 0.97
C18:2 (n-6)	Linoleic acid	26.88 ± 2.46	30.66 ± 2.02	30.82 ± 0.44	30.38 ± 2.00	30.66 ± 0.30	38.84 ± 2.31
C18:3 (n-6)	gamma-Linolenic acid	20.73 ± 1.16	4.58 ± 0.19	4.72 ± 0.21	3.80 ± 0.01	3.16 ± 0.31	2.16 ± 0.43
C18:3 (n-3)	alpha-Linolenic acid [ALA]	n.d	31.54 ± 0.48	35.07 ± 0.69	34.02 ± 1.03	31.57 ± 1.97	20.49 ± 2.11
C20:0	Arachidic acid	0.67 ± 0.04	0.76 ± 0.03	0.71 ± 0.01	0.74 ± 0.06	0.70 ± 0.06	0.52 ± 0.01
C20:1 (n-9)	Cis-11-Eicosenoic acid	0.17 ± 0.01	0.50 ± 0.06	0.23 ± 0.04	0.21 ± 0.01	0.19 ± 0.01	0.04 ± 0.01
C20:2	Cis-11,14-Eicosadienoic acid	0.11 ± 0.02	0.51 ± 0.03	0.28 ± 0.03	0.30 ± 0.01	0.25 ± 0.04	0.10 ± 0.01
C20:3 (n-6)	Cis-8,11,14-Eicosatrienoic acid	n.d	n.d	0.74 ± 0.42	0.47 ± 0.22	0.29 ± 0.37	1.61 ± 1.01
C20:3 (n-3)	Cis-11,14,17-Eicosadienoic acid	0.26 ± 0.16	0.63 ± 0.06	0.87 ± 0.01	0.87 ± 0.01	0.85 ± 0.12	n.d
C21:0	Heneicosanoic acid	n.d	n.d	n.d	0.05 ± 0.01	0.04 ± 0.02	0.05 ± 0.01
C22:0	Behenic acid	0.33 ± 0.01	0.52 ± 0.05	0.43 ± 0.02	0.42 ± 0.05	0.38 ± 0.05	0.27 ± 0.02
C23:0	Tricosanoic acid	n.d	n.d	n.d	0.10 ± 0.01	n.d	0.07 ± 0.01
C24:0	Lignoceric acid	0.23 ± 0.01	0.35 ± 0.06	0.32 ± 0.02	0.34 ± 0.03	0.31 ± 0.05	0.18 ± 0.02

Value(%) in mean ± SE; SFA: saturated fatty acids; MUFA: mono unsaturated fatty acids; PUFA: poly unsaturated fatty acids; UI: unsaturation index; DU: degree of unsaturation; AI: Atherogenic index; TI: Thrombogenic index nd: not detected; All experiments were carried out three times, each with three biological replicates

**Fig. 3:**
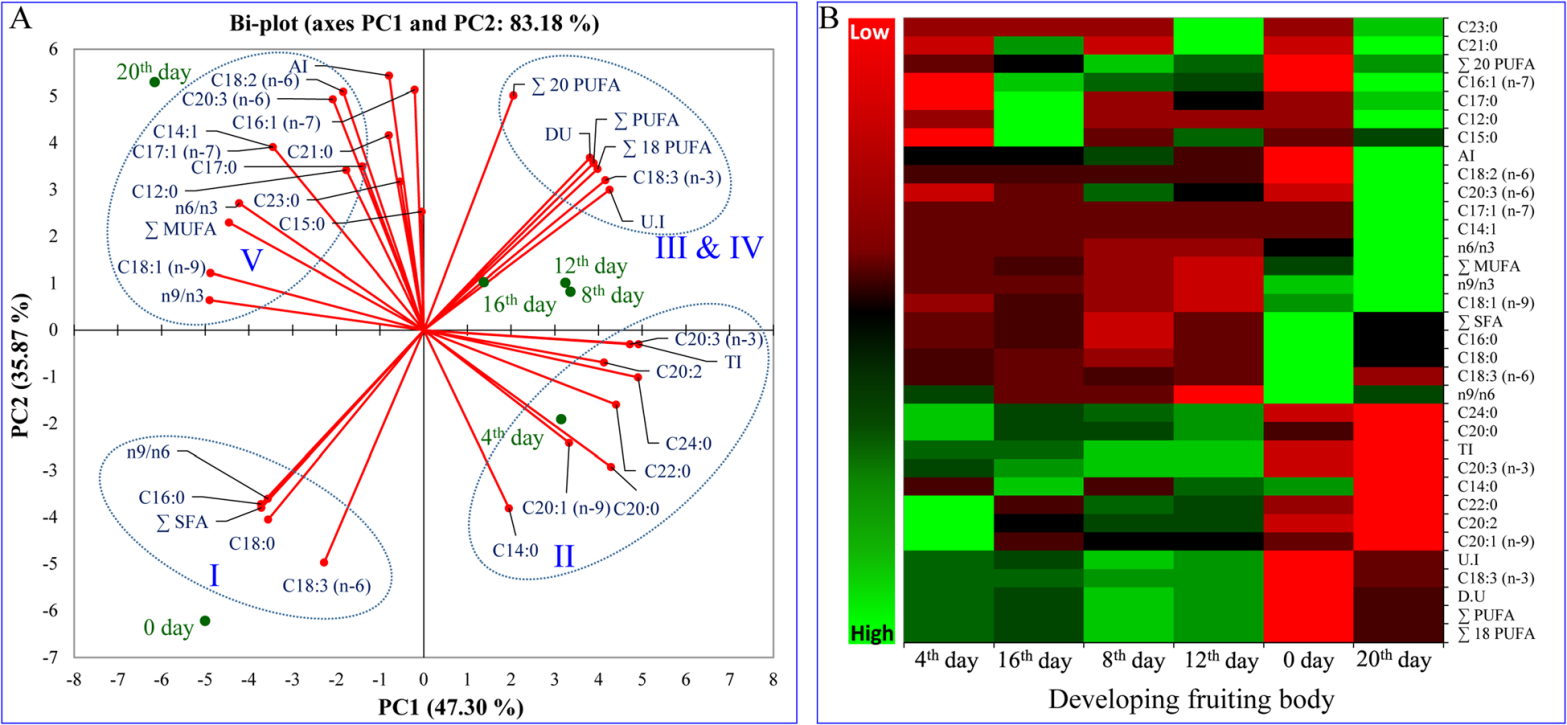
Principal component analysis (PCA) and heat map of total lipids and fatty acids of developing fruit of psyllium. **a** Bi-plot obtained from PCA of data matrix according to Table 1 with first two principal components. **b** Heat map of the differentially synthesised total lipids and fatty acids. All experiments were carried out three times, each with three biological replicate sets and each set contained three replicates. The colors (green to red via black) of the tiles in the heat map represent the values varying from high to low

A range of FAs (C12-C24) were detected, dominanted by linoleic acid (C18:2, n-6; 26–38%), linolenic acid (C18:3, n-3 or n-6; 20–35%) and palmitic acid (C16:0; 20–32%) in the developing fruit of psyllium (Table 1). Five FAs, palmitic acid (C16:0), gamma-linolenic acid (C18:3, n-6), alpha-linolenic acid (C18:3, n-3), stearic acid (C18:0) and *cis*-11-eicosenoic acid (C20:1, n-9), decreased with fruit development. In contrast, linoleic acid (C18:2, n-6) increased with developmental stages and reached a maximum at fruit maturity (38.8%). Four FAs, lauric acid (C12:0), myristoleic (C14:1), *cis*-10-heptadecanoic acid (C17:1, n-7) and tricosanoic acid (C23:0), were detected exclusively at the later stage of development (stage IV and V; 12th–16th and 20th days). Notably, two FAs myristic acid (C14:0) and *cis*-11,14,17-eicosadienoic acid (C20:3, n-3), were found in all stages of fruit development but disappeared at maturity (stage V, 20th day). No remarkable changes were detected in the remaining minor FAs during fruit development (Table 1). Principal component analysis (PCA) indicated that the total lipid and fatty acid contents were significantly correlated with the different developmental stages of the fruit, and the heat map showed the differential fatty acid composition (Fig. 3).

The bi-plot based on PC1 and PC2 showed the possible linkage of FA composition to different stages of development and explained 83.2% of the variation (Fig. 3a). The bi-plot showed that total lipid and FAs were grouped into four clusters. There was a significant shifting of FA composition from bud initiation (stage I: 0 day) to maturity (stage V: 20th day). Overall, PCA exhibited statistically significant differences in lipid and FA composition between developmental stages. There was a significant change from stage I (0 day) to stage III (8th day via stage II, 4th day), but no significant correlation was found from stage III (8th day) to stage IV (12th to 16th days); thereafter a significant change was again noticed from stage IV (12th to 16th days) to stage V (20th day). These observations were also supported by the heat map, which showed significant differences in FA and lipid composition (Fig. 3b).

### Amino acid constituents revealed temporal metabolic pathways

**Fig. 4:**
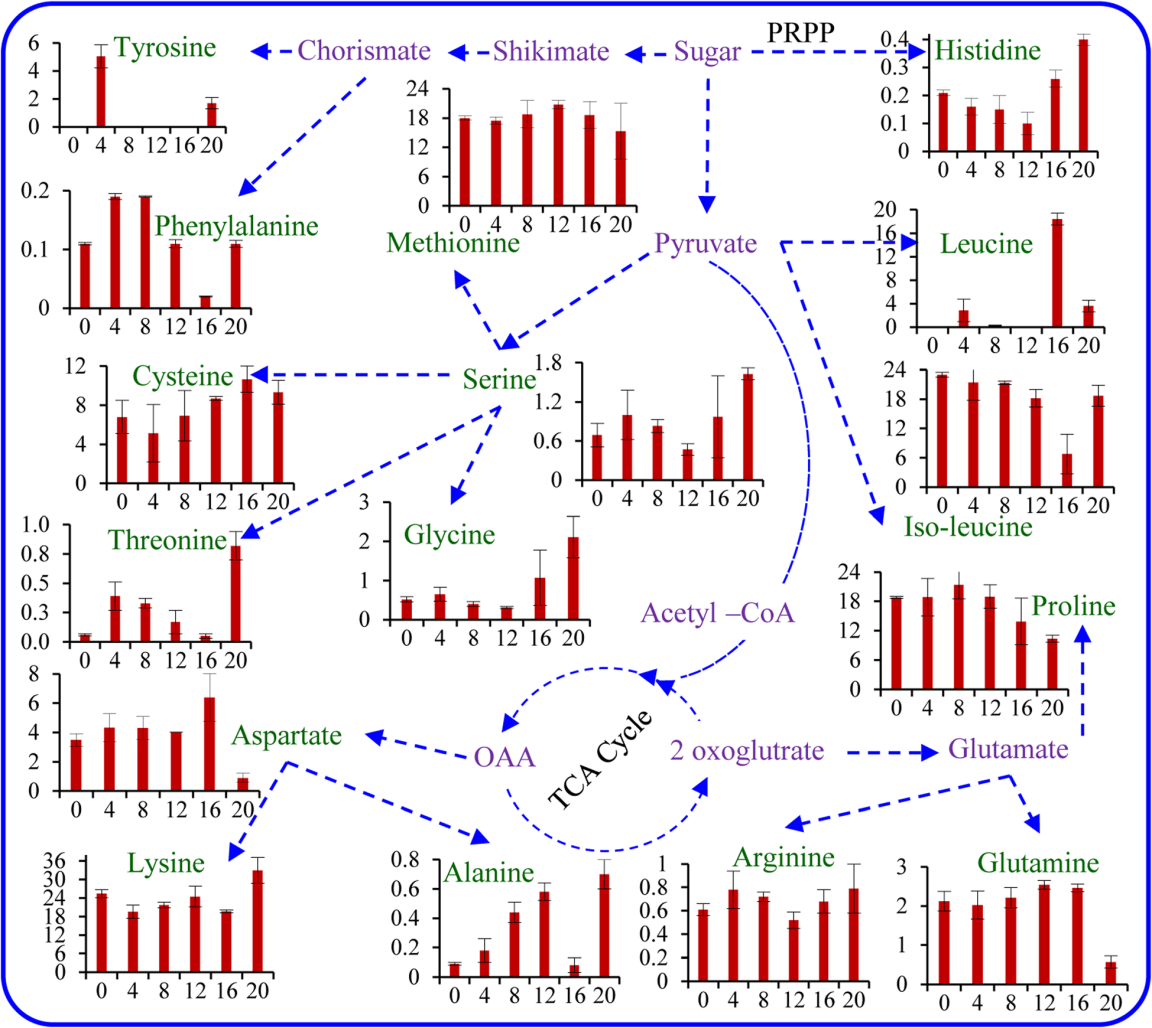
Temporal amino acid pathway inferred in the developing fruit of psyllium. X-axis and Y-axis represent days of fruit development and quantitative mean value (%), respectively as per Table S1. All experiments were carried out three times, each with three biological replicate sets and each set contained three replicates

In total, 16 amino acids were detected, quantified in the different developing stages of psyllium fruit and categorized as non-essential, essential, sulphur-rich and aromatic amino acids (Supplementary Table S1). A similar level of total amino acids was present at various developmental stages, and a possible temporal amino-acid pathway was illustrated (Fig. 4). The essential amino acids, lysine (20–33%) and isoleucine (7–23%) were predominant in the developing psyllium fruit, followed by the non-essential amino acid proline (10–21%) and sulphur-rich methionine (15–21%) and cysteine (5–19%). The non-essential amino acids aspartate, glutamine and glycine were increased with fruit development up to full flowering (stage IV, 12th–16th days); thereafter, a sudden decrease was noticed at maturity (stage V, 20th day). In contrast, an abrupt change in contents of alanine, arginine and serine were observed at different developmental stages, but the maximum levels were detected at maturity. A similar pattern was also noted with the essential amino acids histidine and threonine. However, leucine content was at a maximum at the 16th day of fruit development. The aromatic amino acid phenylalanine was detected in minor quantities, whereas tyrosine was found exclusively at the 4th day and at maturity (i.e. the 20th day). The percent contents of abundant amino acids proline, isoleucine and methionine were decreased at maturity, and in contrast, an elevated amount of lysine was detected.

### Total phenolic and flavonoid contents decrease concomitantly with development of fruit

**Fig. 5:**
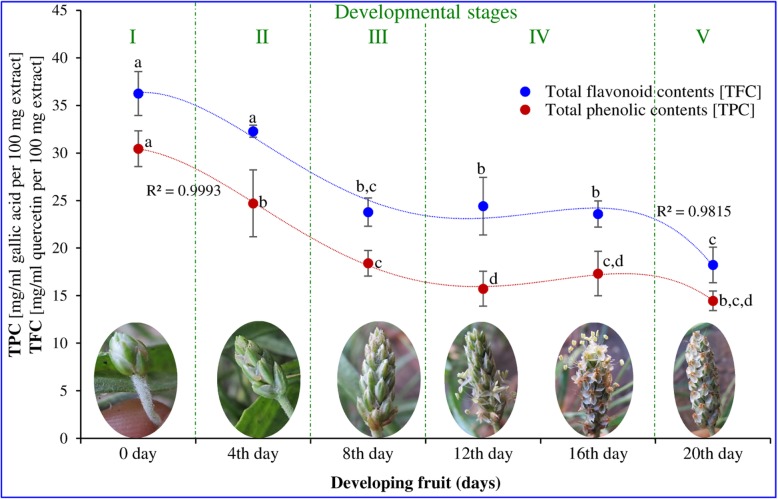
Total flavonoid and total phenolic contents extracted from developing fruiting body of psyllium. All experiments were carried out three times, each with three biological replicate sets and each set contained three replicates. Value represents the mean ± SE and values with different letters are significant different at *p* < 0.05

The highest total phenolic and flavonoid contents were detected at the bud initiation stage, after which they decreased concomitantly with increasing developmental stage and reached a minimum at maturity (Fig. 5). Statistically, a fourth-order polynomial trend line with a regression value about 0.99 clearly demarcated different stages of fruit development and followed a decreasing trajectory path.

### Total antioxidant and scavenging activities of developing fruit

**Fig. 6:**
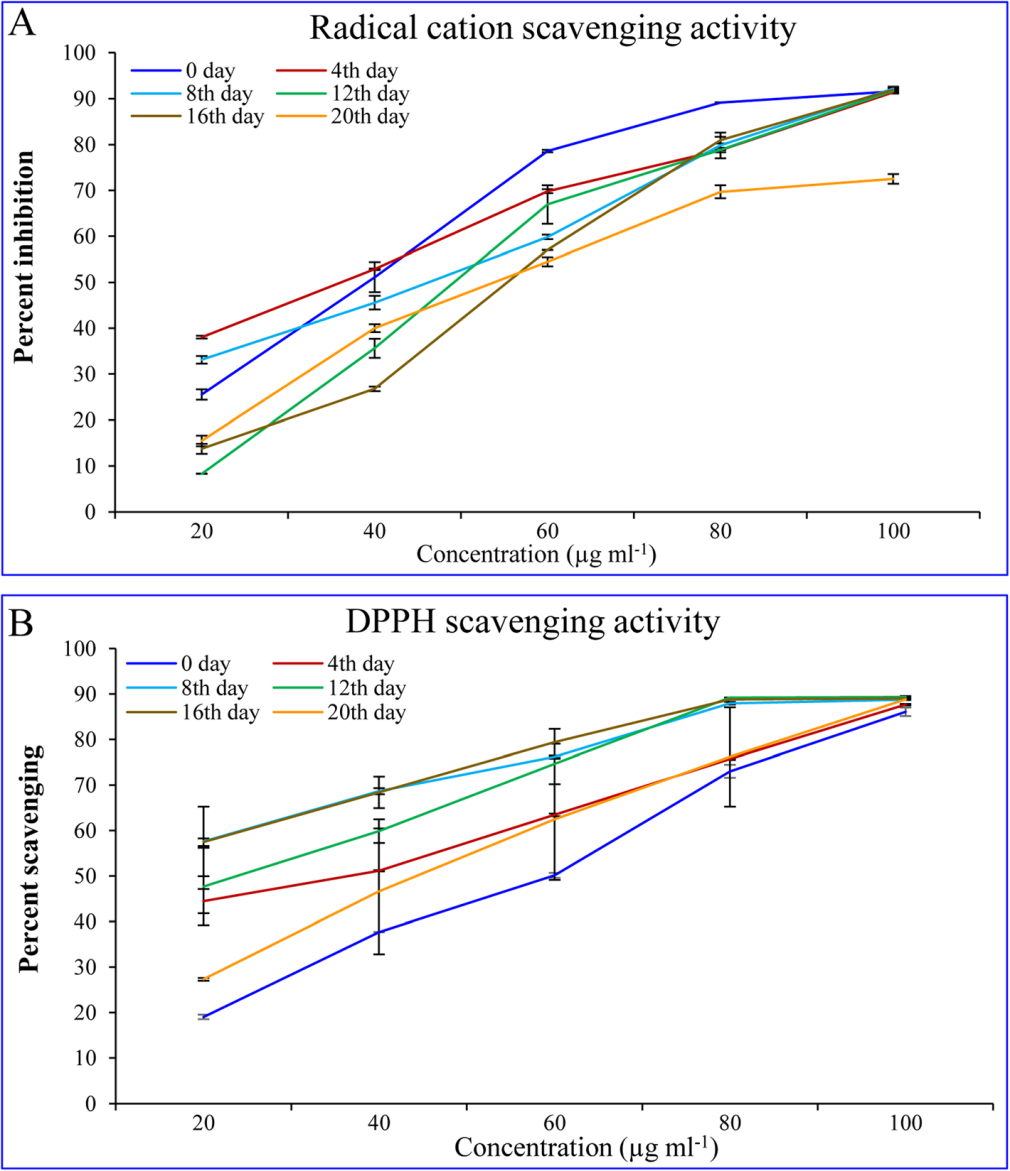
Total antioxidant and scavenging activities of developing fruit of psyllium. **a** Total antioxidant (expressed as the % radical cation ABTS^.+^ scavenging activity) and (**b**) DPPH scavenging activities. All experiments were carried out three times, each with three biological replicate sets and each set contained three replicates. Value represents the mean ± SE

The antioxidant and scavenging activities of the developing fruit extracts increased concomitantly with the extract concentration (Fig. 6). The total antioxidant activity (expressed as the % radical cation ABTS·^+^ scavenging activity) was found to be the highest in stage II (4th day) in a lower extract concentration range (20–40 μg), whereas stage I (0 day) showed maximum activity in a higher extract concentration range (60–100 μg). It was noticed that developing fruit showed similar activity (94%) up to stage IV (12th–16th days), after which a lower activity (72%) was observed with mature fruit extract at the maximum concentration (100 μg) used in the study (Fig. 6a). The maximum DPPH scavenging activity was detected in fruit extract of stage IV (16th day) of development, followed by stage III (8th day) and IV (12th day). About 88% scavenging activity was observed with 100 μg extract of all developing fruiting bodies (Fig. 6b).

### Metabolite profiling of developing circumscissile capsule

**Fig. 7:**
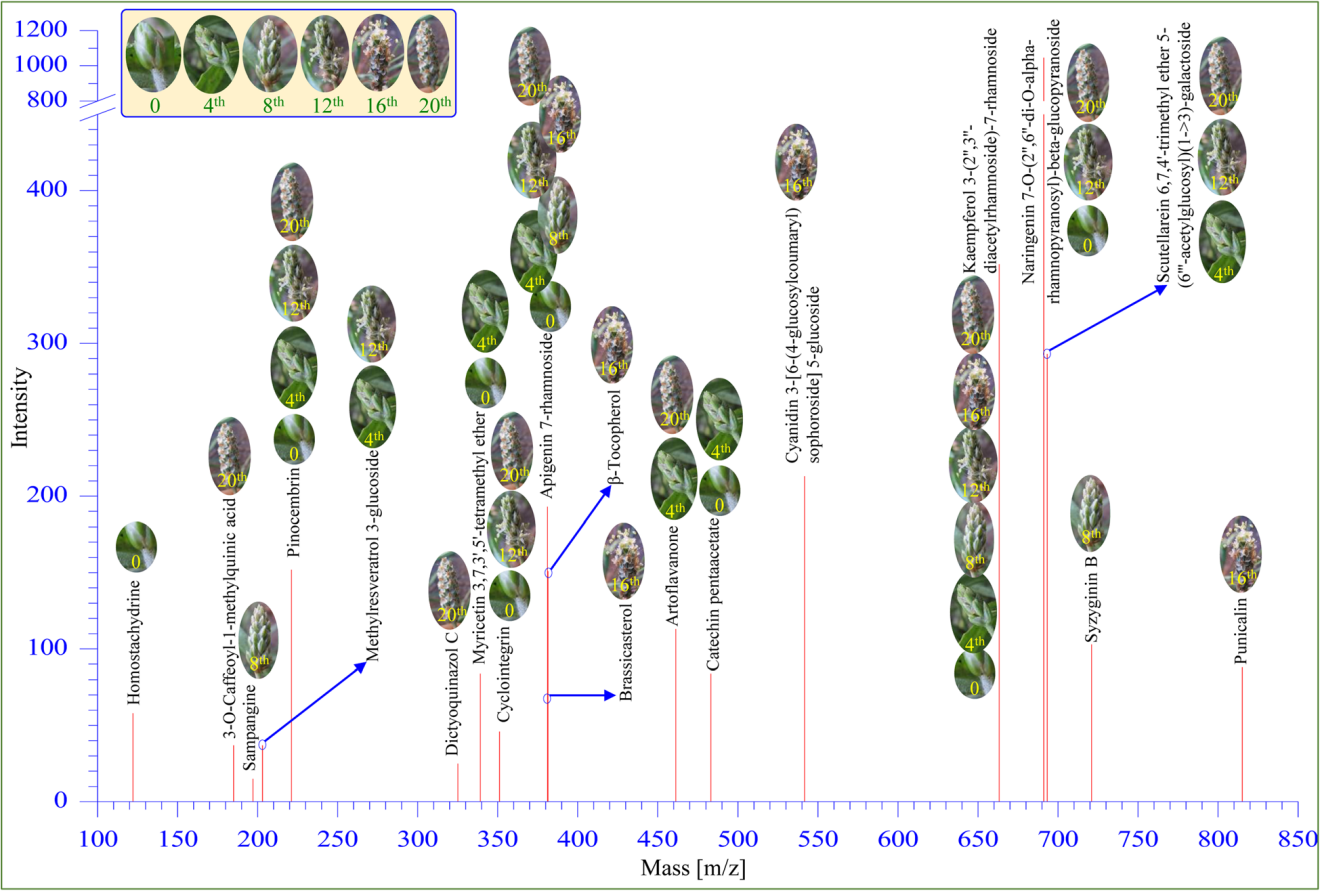
Metabolites detected in developing fruit of psyllium. Each peak (m/z) represents identified metabolites. Images on peak show various developmental stages of psyllium fruit in which metabolites were identified

Nineteen different metabolites (Supplementary Table S2) were identified in the various developmental stages of psyllium fruit (Fig. 7). In total, 10 flavonoids and 2 alkaloids with antioxidant properties were detected (Supplementary Table S3). Two flavonoids, apigenin 7-rhamnoside (m/z 381.10) and kaempferol 3-(2″,3″-diacetylrhamnoside)-7-rhamnoside (m/z 663.19), were ubiquitous in all developmental stages. Two metabolites, catechin pentaacetate (m/z 483.13) and myricetin 3,7,3′,5′-tetramethyl ether (m/z 339.09), were detected in the early developmental stages (stage I, 0 day and II, 4th day) only. Similarly, two metabolites, sampangine (m/z 197.05) and syzyginin B (m/z 721.07), were detected in stage III (8th day) only. A total of four metabolites—brassicasterol (m/z 381.35), cyanidin 3-[6-(4-glucosylcoumaryl) sophoroside] 5-glucoside (m/z 541.66), punicalin (m/z 815.1) and β-tocopherol (m/z 381.35)—were detected exclusively in stage IV (16th day) of fruit development. Similarly, two antioxidant metabolites—dictyoquinazol C (m/z 325.1) and 3-O-Caffeoyl-1-methylquinic acid (, m/z 185.061)—were only identified in mature fruit (stage V, 20th day). The remaining metabolites were detected at different levels depending of the fruit developmental stages.

## Discussion

Flowering plants have an enormous diversity of floral organization and widely variable morphological characteristics. Flower development is a highly coordinated phenomenon, and metabolomics of the model plant *Arabidopsis* revealed that distinct metabolic pathways were stimulated at different stages of flower development [37]. Structural changes characteristic of organ development and the developing flower or fruit are attributed to floral bud initiation, floral organ initiation, floral differentiation and growth leading to seed maturity [5].

*Plantago* flowers are actinomorphic, and the phylogenetic position of *Plantago* makes it a model system to study the molecular developmental mechanism correlated with the loss of dorsal-specific CYC-like gene function [50]. Protogynous is the most distinctive characteristic of *Plantago lanceolata* flowering, in which stigmas are exuded and become receptive before the anthers mature. In the present study, the acropetalous type of inflorescence was observed [52], and anthers were developed in stage II (4th day), whereas growth of the gynoecium was detected later at stage III (8th day) (Fig. 1). After maturity (stage V, 20th day), the characteristic psyllium fruit was developed as a circumscissile capsule, which is small, ovate in shape and dehiscing horizontally through its equator (Fig. 2). Commonly, sepals and petals are attached to the capsule, and the mature seed is covered with a mucilage layer containing hilum. The capsule (fruit) is a small pyxidium and covered by the persistent corolla [52].

Different psyllium plant parts, including leaves, seeds and husk, are considered a rich source of essential FAs, i.e. nutritive PUFAs [45], which cannot be synthesized de novo in humans and are thus required to be outsourced from food [16]. In the present study, developing fruits were found to be a rich source of essential ω-3 alpha-linolenic (ALA) and ω-6 linoleic (LA) and gamma-linolenic (GLA) acids. Similar to the development of *Camelina sativa* fruit, most of the fatty acid profiles of the early stages were different to that of mature stages [51]. A bi-plot data matrix revealed a correlation between FAs and different developmental stages (Fig. 3). A significant shift in lipid and FA composition was noticed from one developmental stage to another, similar to that in *Camelina sativa* [51]. A previous bi-plot analysis confirmed statistically significant differences in lipid and FA composition between different plant parts [45].

In the early stage of development, high levels of SFAs were detected which declined at a later stage (maturity). In contrast, an elevated content of PUFAs, including ALA, LA and GLA, were found in the middle stages of development (Table 1). Among the PUFAs, ALA is extensively explored for a wide range of biological activities, such as nootropic and prophylactic effects, including anti-hyperlipidemic, anti-inflammatory, anti-thrombotic and anti-hypertensive effects [16, 23]. A high atherogenicity index (AI) value favours the adhesion of lipids to the cells; in contrast, a lower AI value inhibits aggregation and levels of esterified fatty acid. Similarly, the thrombogenicity index (TI) also indicates a relationship between SFA and UFA with respect to forming clots in the blood vessels. A high value promotes clotting, whereas a lower value leads to an anti-thrombogenic (anti-clotting) property.

There are many naturally occurring biologically active peptides with the potential to be used in drug development [19]. In total, 16 primary metabolites (amino acids) were identified and quantified in the different developmental stages of psyllium fruit (Supplementary Table S1). The amino acid level was coincident with fruit development, and revealed a possible temporal amino-acid pathway (Fig. 4). A spatial occurrence of amino acids was reported from different plants, including Cumin, *Salicornia* and *Plantago* spp. [33, 39, 45]. Similar to the observation in developing psyllium fruit, some amino acids increased, whereas others decreased at early stages of tomato development; on the other hand, an increase in certain amino acids was found during strawberry and peach development [6, 15, 30]. All developmental stages were dominated by the essential amino acids lysine (20–33%) and isoleucine (7–23%). Similarly, sulphur-rich methionine (15–21%) and cysteine (5–19%) were abundant compared with other amino acids. Similarly, the levels of methionine in tomato and strawberry did not change significantly during development of fruit and ripening [38, 64]. Interestingly, the non-essential amino acids aspartate, glutamine and glycine were increased with fruit development but finally declined at maturity, whereas alanine, arginine, serine, histidine and threonine were detected at the highest levels in the maturity stage. Major amino acids serine, glycine, glutamate, glutamine and leucine were detected throughout seed development in *A. thaliana* [3]. The amino acids alanine, glycine, serine, leucine, lysine, threonine and cysteine were specifically accumulated during later stages of development (stages IV and V; 12th–16th and 20th days), and it was hypothesised that these are involved in protein biosynthesis. Additionally, they may serve as precursors to a number of important metabolic pathways.

Among the natural antioxidants, widely distributed plant flavonoids and phenolic constituents merit emphasis because of their involvement in cellular plant defence systems and are therefore considered as potent antioxidants [13, 33, 57]. Psyllium plant parts contained higher contents of total flavonoids and total phenols and possessed high antioxidant and scavenging activities [45]. In this study, total phenolic and flavonoid contents were decreased concomitantly with developmental stages of the fruit (Fig. 5). Similarly, concentration-dependent total antioxidant and scavenging activities varied according to the different stages of fruit development (Fig. 6). During different developmental stages of in vitro callus culture of *P. ovata*, total antioxidant and scavenging activities increased concurrently with phenolic and flavonoid contents [56]. These results are in accordance with the scavenging and antioxidant activities of *Plantago* plant parts, and polysaccharide, which showed a direct correlation with phenolic and flavonoid contents [42, 43, 45]. The loading plot of primary metabolites, total flavonoids and phenolic contents confirmed the shift in metabolites from stage I (bud initiation, 0 day) to stage V (maturity, 20th day) through different intermediates (Supplementary Figure S1).

Several metabolic changes were observed during developmental stages of psyllium fruit (Fig. 7 and Supplementary Table S3). Out of 19 identified metabolites (Supplementary Table S2), 4 of them—brassicasterol, cyanidin 3-[6-(4-glucosylcoumaryl) sophoroside] 5-glucoside, punicalin and β-Tocopherol —were detected exclusively during flowering (stage IV, 16th day). Most of the phytosterols identified in vegetable oils. The brassicasterol is a free sterol and it show the natural antioxidant activities in foods [9, 36], whereas 2 antioxidant metabolites—dictyoquinazol C and 3-O-Caffeoyl-1-methylquinic acid (MCGA3)—were detected during maturity (stage V, 20th day) only. Similarly, 3-O-Caffeoyl-1-methylquinic acid have strong free radical scavenging activities and inhibition of lipid peroxidation [27] and it was first time characterized from the bamboo (*Phyllostachys edulis*). Interestingly, two metabolites, catechin pentaacetate and myricetin 3,7,3′,5′-tetramethyl ether, were found particularly in the early stages (stage I, 0 day and II, 4th day). The flavonoids apigenin 7-rhamnoside and kaempferol 3-(2″,3″-diacetylrhamnoside)-7-rhamnoside were detected ubiquitously in all developmental stages. Most secondary metabolites, including kaempferol, catechin, and myricetin, are flavonoids and possess potent antioxidant activities [18, 45]. Other metabolites were differentially detected; however, pinocembrin is detected early and late stages of development. Several studies demonstrated the applications of pinocembrin both in vitro and in vivo and suggested that pinocembrin is a good pharmacological drug with potential antioxidative, anti-inflammatory, antitumor, and antimicrobial properties [48]. Previously, the spatial occurrence of metabolites was observed in the different plant parts of psyllium [45]. Similarly, spatial and developmental combinatorial metabolomics was reported in melon fruit, which revealed an extensive metabolic cross-talk [34]. They demonstrated that different developmental stages of a fruit contained a spatial composition of metabolites to which were attributed the organoleptic and nutritional qualities of the fruit [34].

## Conclusion

Previously, a notable shifting of a complex network of metabolites and proteins was established for the developmental processes [5, 30]. In the present study, a time-dependent comparative study of metabolomics, including profiling of primary metabolites (FAs and amino acids), secondary metabolites, total flavonoids and total phenolic content, revealed that fruit development follows a diverse metabolic program. Different biochemical processes and activities (antioxidant and scavenging) undergo a progressive shift along with developmental stages and show a synchronised linkage with relevant metabolites. The availability of important long-chain PUFAs, such as LA, ALA, GLA and AA, have various biomedical and nutraceutical applications. To the best of our knowledge, this is the first study on the metabolic profiles and scavenging activities of developing psyllium fruiting body. Overall, it was concluded that developing psyllium fruiting bodies are a potential candidate to be explored further for nutraceutical applications. Further studies will focus on the validation of identified metabolites, and their integration with transcriptomics data, which will reveal the metabolic regulatory network of psyllium fruit development.

## Methods

### Plant material and sample collection

Seeds of *Plantago ovata* variety IS-3, procured from Seed Spices Research Station, Jagudan, Mehsana, Gujarat, India were sown in an open field (two plots containing garden soil, and each had a dimension of 63 × 36 in.), and plants were grown under natural agro-climatic field conditions [45]. Each plot comprised of eight rows and each row contained about eight plants. Psyllium crops were maintained as per standard commercial practices [24]. Psyllium fruiting bodies were harvested at different developmental stages (0–20 days), and stored in liquid nitrogen for metabolomics and ROS scavenging studies, whereas for morphological studies, samples were stored in FAA (formaldehyde (40%): acetic acid: aqueous ethanol (70%); 5:5:90, v/v/v).

### Eidonomy and anatomy studies

Floral development was studied morphologically with simple photography (DSLR, EOS760D, Canon, Japan) and cryo-scanning electron microscope (cryo-SEM, JSM-7600F, Jeol, Japan). The FAA preserved flower buds were dehydrated in ethanol (50 to 100%), cut into 5 μm thickness using microtome (MT-3, Nippon, Japan) and transverse sections were stained with 0.25% safranin. Sections were examined under light microscope (Axio imager M1, Carl Zeiss, Germany). For cry-SEM, FAA preserved samples were dehydrated in ethanol (50 to 100%), frozen and cryosectioned by using attachments of cryo-SEM and analysed. All images were captured under different magnifications. The morphology of mature seed and dehiscence were studied under compound microscope (Olympus SZX2-ILLD, Tokyo, Japan), whereas the transverse section of mature fruiting body was observed with cryo-SEM.

### Primary metabolites profiling

#### Lipid extraction and fatty acid profiling

Total lipids were extracted from preserved samples (1 g biomass) using chloroform–methanol (1:2 v/v) extraction method [41]. Total lipids were converted to corresponding fatty acid methyl esters (FAMEs) by transmethylation [33]. Samples were methylated with NaOH (1% v/v in methanol; 1 ml) in the vessels, followed by incubation at 55 °C for 15 min in a water-bath. After that, methanolic HCl (5% v/v; 2 ml) was added and further heated at 55 °C for 15 min. Deionized water–hexane mixture (1:2; 3 ml) was added to obtain phase separation. The derivative FAMEs were recovered and extracted in hexane (2 ml), dried under N_2_ and dissolved in hexane (200 μl). FAME samples were identified and quantified by gas chromatography coupled with mass spectrometer (GCMS–QP2010, Shimadzu, Japan) equipped with an auto-sampler (AOC-5000) and flame ionization detection (FID)/capillary column using previously optimised methods [33, 39].

Total saturated fatty acids (SFA) and unsaturated fatty acids (monounsaturated fatty acids, MUFA and polyunsaturated fatty acids, PUFA) were quantified by summation of percent quantity of corresponding fatty acids [41]. Unsaturation index (equation 1) [47], degree of unsaturation (equation 2) [63], atherogenic (equation 3) and thrombogenic (equation 4) indices [11] were calculated using the following equations–.

1 $$ Unsaturation\kern0.17em index\;(UI)=\sum \left( UFA\;w\%\times number\kern0.17em of\kern0.17em double\kern0.17em bonds\right) $$

2 $$ Degree\kern0.17em of\kern0.17em unsaturation\;(DU)=\left( MUFA\;w\%\right)+2\times \left( PUFA\;w\%\right) $$

3 $$ Atherogenic\kern0.34em index\kern0.28em (AI)=\frac{C12+C14+C16}{\sum n3 PUFA+\sum n6 PUFA+\sum MUFA} $$

4 $$ Thrombogenic\kern0.17em index\;(TI)=\frac{C14+C16+C18}{\left(0.5\times n6 PUFA\right)+\left(3\times n3 PUFA\right)+\left(n3/n6 PUFA\right)} $$

#### Amino acid profiling

Amino acids were identified and quantified by HPLC system (Waters Alliance model, 2996-separation module with auto-sampler, USA) based on the phenylisothiocyanate (PITC) derivatisation method [26]. Total Protein was extracted from preserved fruiting bodies (1 g) using trichloroacetic acid (TCA) precipitation method. The total protein of each sample was hydrolysed by HCl (6N, 500μl) in a sealed glass vessel by heating at 110°C for 24h in an oven. After hydrolysis, vessels were broken and protein samples were vacuum dried in a desiccator. Protein samples and amino acid standard (AAS18, Sigma, St. Louis, Missouri, USA) were neutralised by adding the mixture of ethanol–water–TEA (triethylamine) (2:2:1 v/v/v; 500μl). Samples were derivatised by adding a mixture of ethanol–water–TEA–PITC (7:1:1:1 v/v/v/v; 500μl). The reaction mixture was vortexed properly, kept at room temperature for 20min and vacuum dried. Dried samples were dissolved in Na_2_HPO_4_ buffer (400μl; 5mM, pH 7.4) containing acetonitrile (5% v/v). Each sample was filtered with a 0.2 μm membrane and amino acid composition was analysed using HPLC [32].

### Plant extract preparation

Plant samples (10 g) were ground in liquid N_2,_ transferred to aqueous methanol (70%, v/v) and kept overnight for extraction. The supernatant was collected by centrifugation at 8000 rpm for 10 min. The plant extracts were concentrated in a rotary evaporator (150–100 mbar at 37 °C), lyophilised and stored at − 20 °C for further study.

To determine total phenolic content, flavonoid content, antioxidant activities, and scavenging activities, the absorbance reading of samples (plant extracts) were compared with a standard curve, which was drawn by the same method, using known amount of the corresponding standard. All tests were performed in triplicates, each containing three biological replicates and values were expressed as mean ± SE.

### Determination of total phenolic and total flavonoid contents

Total phenolic content of the extract was determined by Folin-Ciocalteu (FC) reagents (Sigma–Aldrich, USA) using gallic acid as a standard [22, 33]. Folin–Ciocalteu reagent (2.5 ml of 0.2 N) was mixed with extract, incubated for 5 min, after that reaction gets neutralized by adding 2 ml sodium carbonate (Na_2_CO_3;_ 75 g l^− 1^). The reaction mixtures were incubated further for 90 min at room temperature and absorbance was taken at 760 nm. Total phenolic content was calculated as mg ml^− 1^ gallic acid per 100 mg of extract from a standard curve.

To determine the total flavonoid content, 0.3 ml NaNO_2_ (5%, v/v) was added to the extract and mixture was incubated for 5 min at room temperature followed by addition of 0.3 ml AlCl_3_ (10%, v/v) and 2 ml NaOH (1 M). The reaction mixture was diluted with water and absorbance was recorded at 510 nm. The total flavonoid content was calculated as mg ml^− 1^ quercetin per 100 mg of extract from a standard curve [57, 66].

### Total antioxidant and scavenging activity

Total antioxidant activity of plant extracts were measured by comparing ABTS^.+^ (2, 2′-azinobis-(3-ethylbenzothiazoline-6-sulfonic acid) radical cation scavenging capability [22, 49, 58]. The ABTS free radical cations were generated by mixing ABTS diammonium salt (7 mM) solution with potassium persulfate (2.45 mM) followed by incubation at room temperature for 12–16 h in the dark. The initial absorbance of ABTS^.+^ solution (at 735 nm) was adjusted to 0.70 ± 0.02 by diluting with water. Diluted ABTS^.+^ solution (1 ml) was mixed with a different concentration of the plant extracts or standard (1–5 μg ml^− 1^ trolox). The absorbance was recorded at 735 nm after incubation; percentage inhibition was calculated and free radical cation scavenging activity was measured by comparing with trolox, which was used as a standard.

DPPH (2, 2-diphenyl-1-picrylhydrazyl) is a free radical, which turns from deep violet to pale yellow non-radical form of DPPH-H, due to the radical scavenging activity of antioxidants. The scavenging assay was performed by measuring change in the colour of DPPH solution [53]. The initial absorbance of DPPH stock solution (0.024% w/v in methanol) was adjusted about 0.98 ± 0.02 at 517 nm by diluting with methanol. DPPH working solution (3 ml) was mixed with a different concentration of extracts and incubated at room temperature in the dark for overnight. The absorbance was recorded at 517 nm and DPPH radical scavenging activity was estimated using the equation 5.

5 $$ Scavenging\kern0.17em activity\;\left(\%\right)=\left[\frac{O{D}_{517}\; of\kern0.17em Control-O{D}_{517}\; of\kern0.17em Extract}{O{D}_{517}\; of\kern0.17em Control}\right]\times 100 $$

### Extraction and identification of secondary metabolites

The each stage of fruiting body (100 mg) was homogenised in liquid N_2_ and metabolites were extracted in ice-cold aqueous methanol (70%, v/v) followed by vortexing [45]. The samples were kept for sonication in an ultrasonic water bath (MRC, Holon, Israel) for one hour (at frequency 40 kHz; 25 °C). The samples were centrifuged (20,000 rpm at 25 °C for 10 min), supernatant was collected and filtered through 0.2 μm membrane. The filtered solution was used for metabolites analysis using LC coupled with TOF MS/MS system (Micromass, Waters, USA) in positive-ion mode [12]. The scanning range was 0–1000 m/z with an acquisition rate of 0.25 s and inter-scan delay of 0.1 s. The background of each spectrum was subtracted, the data smoothed, centered and peaks were integrated, using the Mass Lynx software version 4.1 (Micromass, Waters, USA). Metabolites were identified by comparing LC-TOF-MS/MS peaks using on-line database [67].

### Statistical analysis

All experiments were carried out three times, each with three biological replicate sets and each set contained three replicates (i.e. total 27 samples were used for the study in 3 × 3 × 3 pattern). Data for the each experiment was subjected to analysis of variance (ANOVA) to determine differences and expressed as the mean ± SE (standard error of the mean). Statistical significance was determined at *p ≤ 0.05* using Tukey’s multiple comparison method and mean values that were significantly different within a treatment are indicated by different letters. Total lipid composition was statistically analysed by principal component analysis (PCA) and a heat map was generated. Lipid data set was used to generate Pearson’s correlation matrix, and PCA along with respective heat map was inferred [55, 61] using different software including Sigma Plot (ver. 12), SYSTAT (ver. 13) and Origin (ver. 15).

## Supplementary information

**Additional file 1: Table S1.** Amino-acid composition of developing fruiting body of psyllium.

**Additional file 2: Table S2.** Basic chemical structure of metabolites identified in developing fruits of psyllium.

**Additional file 3: Table S3.** Probable metabolites identified by LC-MS (+ve mode) in developing fruit of psyllium.

**Additional file 4: Figure S1.** Loading plot of PC analysis (PCA) based on (A) fatty acid composition, (B) amino-acids and (C) total flavonoid and phenolic contents of developing psyllium fruit.

## Data Availability

The datasets used and/or analysed during the current study available from the corresponding author on reasonable request.

## Abbreviations

ABTS: 2, 2′-azinobis-(3-ethylbenzothiazoline-6-sulfonic acid)
AI: Atherogenicity index
ALA: Alpha-linolenic acid
ANOVA: Analysis of variance
DPPH: 2, 2-diphenyl-1-picrylhydrazyl
DU: Degree of unsaturation
FA: Fatty acid
GC-MS: Gas chromatography-mass spectrometry
GLA: Gamma-linolenic acid
HPLC: High-performance liquid chromatography
LA: Linoleic acid
LC-MS: Liquid chromatography-mass spectrometry
MUFA: Mono unsaturated fatty acids
PCA: Principal component analysis
PUFA: Poly unsaturated fatty acids
ROS: Reactive oxygen species
SEM: Scanning electron microscope
SFA: Saturated fatty acids
TI: Thrombogenicity index
UI: Unsaturation index
