# Supplementary material for: Metabolic profiling and scavenging activities of developing circumscissile fruit of psyllium (Plantago ovata Forssk.) reveal variation in primary and secondary metabolites
Source: BMC Plant Biol. 2020 Mar 14;20:116. doi: 10.1186/s12870-020-2318-5 (PMC7071626; doi:10.1186/s12870-020-2318-5)
Supplement: Supplementary file 1 — Additional file 1: Table S1. Amino-acid composition of developing fruiting body of psyllium. [file 12870_2020_2318_MOESM1_ESM.pdf]

Table S1: Amino-acid composition of developing fruiting body of psyllium

Amino acids	0 day	4th day	8th day	12th day	16th day	20th day
Non-essential						
Alanine	0.09±0.01	0.18±0.08	0.44±0.07	0.58±0.46	0.08±0.05	0.70±0.33
Arginine	0.61±0.05	0.78±0.26	0.72±0.04	0.52±0.07	0.68±0.36	0.79±0.69
Aspartate	3.49±0.43	4.33±0.94	4.3±0.79	4.01±0.02	6.38±2.47	0.90±0.32
Glutamine	2.12±0.25	2.02±0.36	2.21±0.26	2.54±0.11	2.46±0.10	0.57±0.15
Glycine	0.52±0.07	0.65±0.18	0.40±0.06	0.31±0.03	1.07±0.71	2.11±0.53
Proline	18.8±0.22	18.86±3.84	21.36±2.82	18.98±2.41	13.89±4.75	10.36±0.71
Serine	0.69±0.18	1.00±0.38	0.83±0.10	0.47±0.09	0.97±0.63	1.63±0.09
Essential						
Histidine	0.21±0.01	0.16±0.03	0.15±0.05	0.10±0.04	0.26±0.03	0.40±0.02
Isoleucine	22.98±0.49	21.4±3.62	21.37±0.35	18.19±1.78	6.78±4.06	18.7±2.12
Leucine	0.08±0.01	2.84±3.92	0.26±0.05	0.08±0.02	18.41±1.00	3.61±1.00
Lysine	25.45±1.29	19.53±2.14	21.71±0.92	24.48±3.37	19.65±0.44	32.94±4.22
Threonine	0.06±0.01	0.39±0.32	0.33±0.04	0.17±0.01	0.05±0.02	0.82±0.12
S-rich						
Cysteine	6.80±1.70	5.13±2.95	6.94±2.58	8.7±0.19	18.66±12.93	9.34±1.21
Methionine	17.99±0.45	17.48±0.75	18.79±2.80	20.76±0.85	18.64±2.76	15.32±5.70
Aromatic						
Phenylalanine	0.11±0.02	0.19±0.05	0.19±0.01	0.11±0.07	0.02±0.01	0.11±0.06
Tyrosine	nd	5.06±3.83	nd	nd	nd	1.70±0.04

Value (%) in mean ±SE. All experiments were carried out three times, each with three biological replicates.
