# Supplementary material for: Metabolic profiling and scavenging activities of developing circumscissile fruit of psyllium (Plantago ovata Forssk.) reveal variation in primary and secondary metabolites
Source: BMC Plant Biol. 2020 Mar 14;20:116. doi: 10.1186/s12870-020-2318-5 (PMC7071626; doi:10.1186/s12870-020-2318-5)
Supplement: Supplementary file 2 — Additional file 2: Table S2. Basic chemical structure of metabolites identified in developing fruits of psyllium. [file 12870_2020_2318_MOESM2_ESM.pdf]

Table S2: Basic chemical structure of metabolites identified in developing fruits of psyllium

**Metabolites^**	**IUPAC Name#**	**Structure of Metabolites***
Apigenin 7-rhamnoside	5-hydroxy-2-(4-hydroxyphenyl)-7-[(2S,4S,5R)-3,4,5-trihydroxy-6-methyloxan-2-yl]oxychromen-4-one	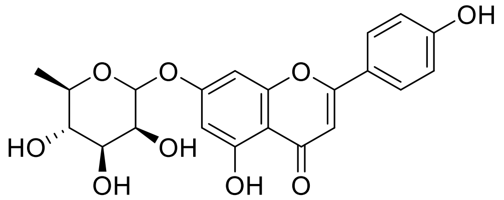
Artoflavanone	5-hydroxy-7-methoxy-6-(3-methylbut-2-enyl)-2-(3,4,5-trimethoxyphenyl)-2,3-dihydrochromen-4-one	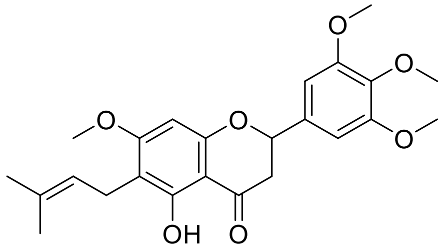
Brassicasterol	(3S,8S,9S,10R,13R,14S,17R)-17-[(E,2R,5R)-5,6-dimethylhept-3-en-2-yl]-10,13-dimethyl-2,3,4,7,8,9,11,12,14,15,16,17-dodecahydro-1H-cyclopenta[a]phenanthren-3-ol	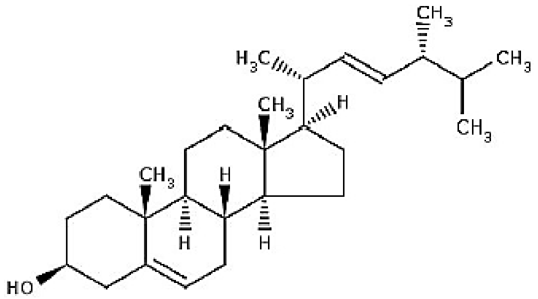
Catechin pentaacetate	[(2R,3S)-5,7-diacetyloxy-2-(3,4-diacetyloxyphenyl)-3,4-dihydro-2H-chromen-3-yl] acetate	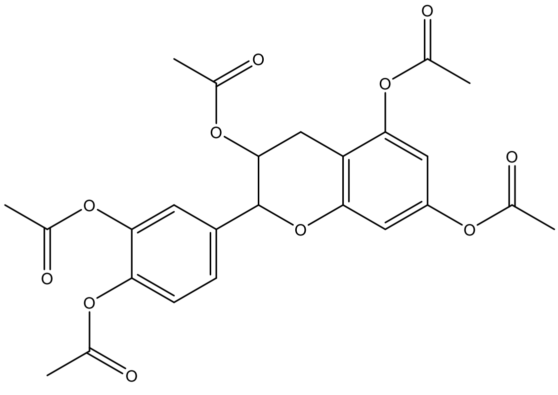
Cyanidin 3-[6-(4-glucosylcoumaryl) sophoroside] 5-glucoside	[6-[2-(3,4-dihydroxyphenyl)-7-hydroxy-5-[3,4,5-trihydroxy-6-(hydroxymethyl)oxan-2-yl]oxychromenylium-3-yl]oxy-3,4-dihydroxy-5-[3,4,5-trihydroxy-6-(hydroxymethyl)oxan-2-yl]oxyoxan-2-yl]methyl (E)-3-[4-[3,4,5-trihydroxy-6-(hydroxymethyl)oxan-2-yl]oxyphenyl]prop-2-enoate	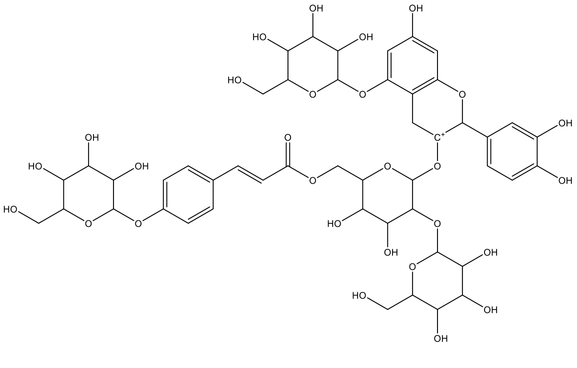

Cyclointegrin	5,15-dihydroxy-17-methoxy-9,9-dimethyl-8,20-dioxatetracyclo[10.8.0.0^{2,7}.0^{14,19}]icosa-1(12),2(7),3,5,14,16,18-heptaen-13-one	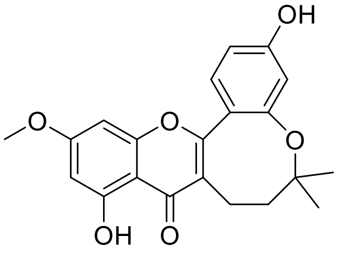
Dictyoquinazol C	3-[2-(hydroxymethyl)-4-methoxyphenyl]-6-methoxy-4-oxo-2*H*-quinazoline-1-carbaldehyde	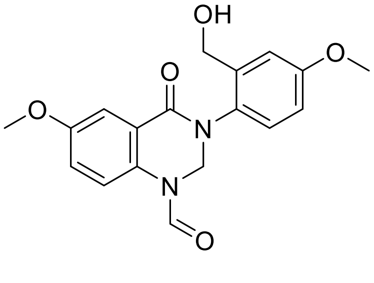
Homostachydrine	(2S)-1,1-dimethylpiperidin-1-ium-2-carboxylate	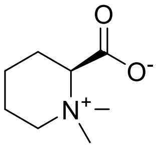
Kaempferol 3-(2",3"-diacetylrhamnoside)-7-rhamnoside	[(2S,3S,5S)-3-acetyloxy-5-hydroxy-2-[5-hydroxy-2-(4-hydroxyphenyl)-4-oxo-7-[(2S,3S,5R)-3,4,5-trihydroxy-6-methyloxan-2-yl]oxychromen-3-yl]oxy-6-methyloxan-4-yl] acetate	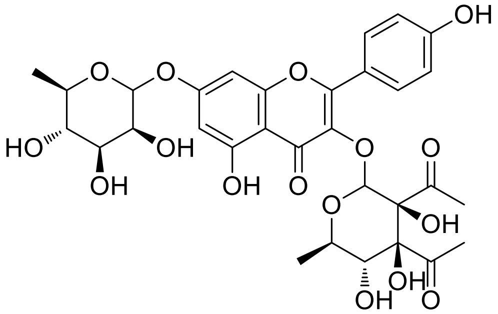
3-O-Caffeoyl-1-methylquinic acid	(1S,3R,4R,5R)-3-[(E)-3-(3,4-dihydroxyphenyl)prop-2-enoyl]oxy-4,5-dihydroxy-1-methoxycyclohexane-1-carboxylic acid	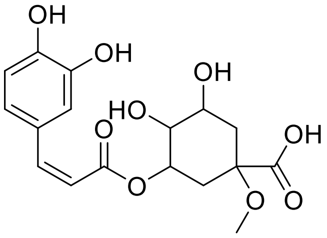
Methylresveratrol 3-glucoside	2-[3-hydroxy-5-[(Z)-2-(4-methoxyphenyl)ethenyl]phenoxy]-6-(hydroxymethyl)oxane-3,4,5-triol	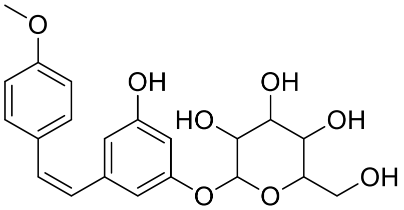

Myricetin 3,7,3',5'-tetramethyl ether	5-hydroxy-2-(4-hydroxy-3,5-dimethoxyphenyl)-3,7-dimethoxychromen-4-one	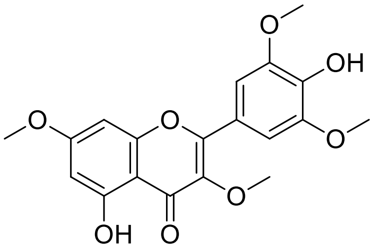
Naringenin 7-O-(2",6"-di-O-alpha-rhamnopyranosyl)-beta-glucopyranoside)	7-[(2S,4S,5S)-4,5-dihydroxy-3-[(2R,3S,5R)-3,4,5-trihydroxy-6-methyloxan-2-yl]oxy-6-[[(2R,3S,5R)-3,4,5-trihydroxy-6-methyloxan-2-yl]oxymethyl]oxan-2-yl]oxy-5-hydroxy-2-(4-hydroxyphenyl)-2,3-dihydrochromen-4-one	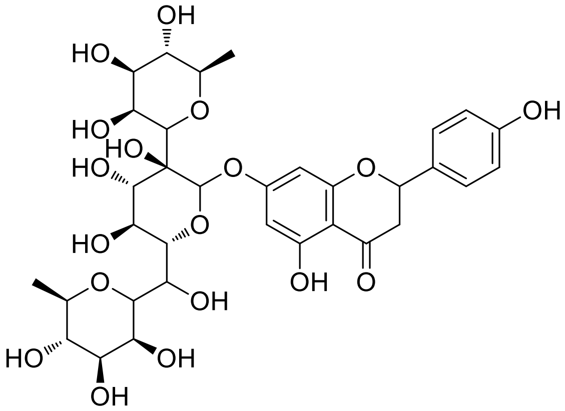
Pinocembrin	5,7-dihydroxy-2-phenyl-2,3-dihydrochromen-4-one	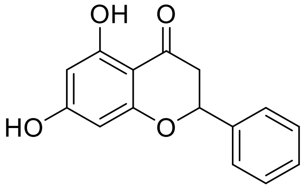
Punicalin	3,4,5,11,12,13,21,22,23,26,27,38,39-tridecahydroxy-9,14,17,29,36-pentaoxaoctacyclo[29.8.0.$0^{2,7}$.$0^{10,15}$.$0^{19,24}$.$0^{25,34}$.$0^{28,33}$.$0^{32,37}$]nonatriaconta-1(39),2,4,6,19,21,23,25,27,31,33,37-dodecaene-8,18,30,35-tetrone	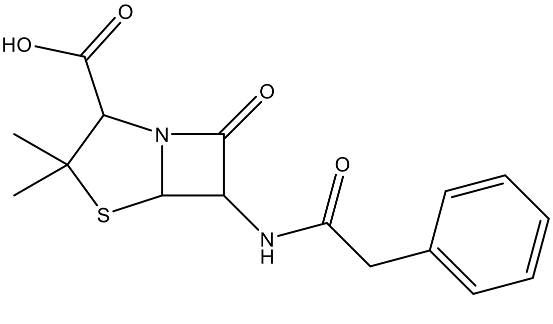
Sampangine	10,16-diazatetracyclo[7.7.1.$0^{2,7}$.$0^{13,17}$]heptadeca-1(16),2,4,6,9,11,13(17),14-octaen-8-one	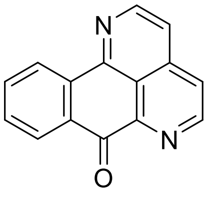

Scutellarein 6,7,4'-trimethyl ether 5-(6'''-acetylglucosyl)(1->3)-galactoside	[(3S,4S,6S)-6-[(2S,4S,5S)-2-[6,7-dimethoxy-2-(4-methoxyphenyl)-4-oxochromen-5-yl]oxy-6-ethyl-3,5-dihydroxyoxan-4-yl]oxy-3,4,5-trihydroxyoxan-2-yl]methyl acetate	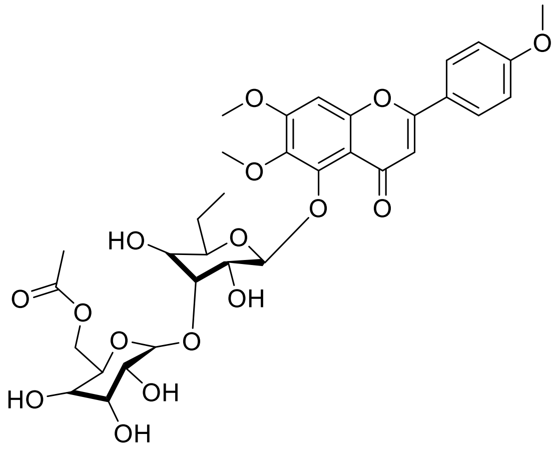
Syzyginin B	[(10R,11R,12R,13R,15R)-3,4,5,12,13,21,25,26,27-nonahydroxy-8,18-dioxo-9,14,17,23,30-pentaoxahexacyclo[17.12.0.02,7.010,15.022,31.024,29]hentriaconta-1(31),2,4,6,19,21,24(29),25,27-nonaen-11-yl] 3,4,5-trihydroxybenzoate	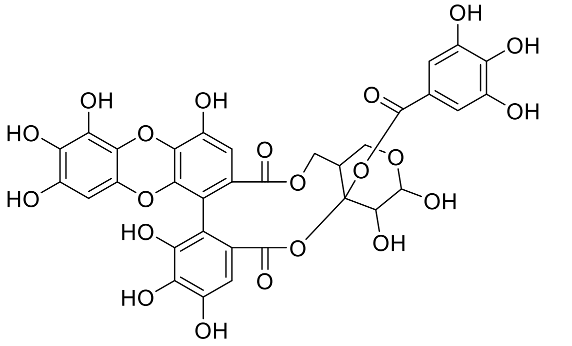
β-Tocopherol	(2R)-2,5,8-trimethyl-2-[(4R,8R)-4,8,12-trimethyltridecyl]-3,4-dihydrochromen-6-ol	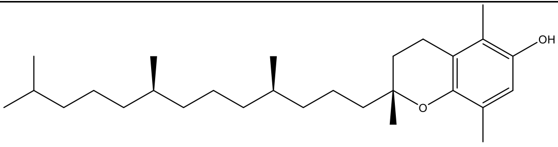

^ Probable metabolites identified by LCMS (+ve mode) using METLIN database (Table S3)

#Source: PubChem; URL: https://pubchem.ncbi.nlm.nih.gov (computed by PubChem; LexiChem 2.6.6)

*Redrawn by Chem Draw
