# Supplementary material for: Metabolic profiling and scavenging activities of developing circumscissile fruit of psyllium (Plantago ovata Forssk.) reveal variation in primary and secondary metabolites
Source: BMC Plant Biol. 2020 Mar 14;20:116. doi: 10.1186/s12870-020-2318-5 (PMC7071626; doi:10.1186/s12870-020-2318-5)
Supplement: Supplementary file 3 — Additional file 3: Table S3. Probable metabolites identified by LC-MS (+ve mode) in developing fruit of psyllium. [file 12870_2020_2318_MOESM3_ESM.pdf]

Table S3: Probable metabolites identified by LCMS (+ve mode) in developing fruit of psyllium

**Mol id**	**Input m/z**	**Expected Mass**	**∆ppm**	**Possible adducts**	**Probable Metabolites**	**0 day**	**4th day**	**8th day**	**12th day**	**16th day**	**20th day**	**Remarks**	**References**
48773	381.10	416.1107	5	[M+H-2H2O]+	Apigenin 7-rhamnoside	√	√	√	√	√	√	Flavonol	Ahmed et al., 1987
87229	461.22	428.1835	6	[M+CH3OH+H]+	Artoflavanone	nd	√	nd	nd	nd	√	Flavanone	Dayal and Seshadri, 1974
3519	381.35	398.3548	5	[M+H-H2O]+	Brassicasterol	nd	nd	nd	nd	√	nd	Sterol	Mo et al., 2013
44485	483.13	500.1319	1	[M+H-H2O]+	Catechin pentaacetate	√	√	nd	nd	nd	nd	Flavonol	Murphy et al., 2003
95582	541.66	1081.3036	1	[M+2H]2+	Cyanidin 3-[6-(4-glucosylcoumaryl) sophoroside] 5-glucoside	nd	nd	nd	nd	√	nd	Flavonoid glycosides	Yannai, 2003
49963	351.12	368.1260	9	[M+H-H2O]+	Cyclointegrin	√	nd	nd	√	nd	√	Flavonol	Harborne et al., 2013
94945	325.12	342.1216	3	[M+H-H2O]+	Dictyoquinazol C	nd	nd	nd	nd	nd	√	Alkaloid	Lee et al., 2002
66883	122.097	157.1103	4	[M+H-2H2O]+	Homostachydrine	√	nd	nd	nd	nd	nd	Betaine	Wiehler and Marion, 1958
50374	663.19	662.1847	2	[M+H]+	Kaempferol 3-(2",3"-diacetylrhamnoside)-7-rhamnoside	√	√	√	√	√	√	Flavonol	Min et al., 2001
94501	185.061	368.1107	8	[M+2H]2+	3-O-Caffeoyl-1-methylquinic acid	nd	nd	nd	nd	nd	√	Antioxidant	Kweon et al. 2001
89724	203.08	404.1471	4	[M+2H]2+	Methylresveratrol 3-glucoside	nd	√	nd	√	nd	nd	Stilbene glycoside	Yannai, 2003
51196	339.09	374.1002	7	[M+H-2H2O]+	Myricetin 3,7,3',5'-tetramethyl ether	√	√	nd	nd	nd	nd	Flavonoid	Harborne and Baxter, 1999
52750	691.22	726.2371	6	[M+H-2H2O]+	Naringenin 7-O-(2",6"-di-O-alpha-rhamnopyranosyl)-beta-glucopyranoside	√	nd	nd	√	nd	√	Flavonol	Nakagawa et al., 2006
44418	221.06	256.0736	3	[M+H-2H2O]+	Pinocembrin	√	√	nd	√	nd	√	Flavanone	Rasul et al., 2013
93797	815.1	782.0602	7	[M+CH3OH+H]+	Punicalin	nd	nd	nd	nd	√	nd	Ellagitannin	Zhang et al., 2009
89826	197.05	232.0637	4	[M+H-2H2O]+	Sampangine	nd	nd	√	nd	nd	nd	Alkaloid	Rao et al., 1986
49567	693.24	692.2316	1	[M+H]+	Scutellarein 6,7,4'-trimethyl ether 5-(6'"-acetylglucosyl)(1->3)-galactoside	nd	√	nd	√	nd	√	Flavonol	Harborne and Baxter, 1999
86823	721.07	756.0810	2	[M+H-2H2O]+	Syzyginin B	nd	nd	√	nd	nd	nd	Tannin	Tanaka et al., 1996
230	381.35	416.3654	7	[M+H-2H2O]+	β-Tocopherol	nd	nd	nd	nd	√	nd	Vitamin	Demo et al., 1998

nd: not detected and √: present (detected)

References

Ahmed, A.A., El-Sayed, N.H., el-Negoumy, S.I. and Mabry, T.J., 1987. Flavonoids of *Cotula cinerea*. Journal of Natural Products, 50(3), pp.519-520.

Dayal, R. and Seshadri, T.R., 1974. Colourless components of the roots of *Artocarpus heterophyllus*: Isolation of a new compound, artoflavanone. Indian Journal of Chemistry.

Demo, A., Petrakis, C., Kefalas, P. and Boskou, D., 1998. Nutrient antioxidants in some herbs and Mediterranean plant leaves. Food Research International, 31(5), pp.351-354.

Harborne, J.B. and Baxter, H., 1999. The handbook of natural flavonoids. Volume 1 and Volume 2. John Wiley and Sons.

Harborne, J.B., Marby, H. and Marby, T.J., 2013. The flavonoids. Springer.

Kweon, M.H., Hwang, H.J. and Sung, H.C., 2001. Identification and antioxidant activity of novel chlorogenic acid derivatives from bamboo (*Phyllostachys edulis*). Journal of Agricultural and Food Chemistry, 49(10), pp.4646-4655.

Lee, I.K., Yun, B.S., Han, G., Cho, D.H., Kim, Y.H. and Yoo, I.D., 2002. Dictyoquinazols A, B, and C, new neuroprotective compounds from the mushroom Dictyophora indusiata. Journal of Natural Products, 65(12), pp.1769-1772.

Min, B.S., Tomiyama, M., Nakamura, N. and Hattori, M., 2001. Kaempferol acetylrhamnosides from the rhizome of *Dryopteris crassirhizoma* and their inhibitory effects on three different activities of human immunodeficiency virus-1 reverse transcriptase. Chemical and Pharmaceutical Bulletin, 49(5), pp.546-550.

Mo, S., Dong, L., Hurst, W.J. and van Breemen, R.B., 2013. Quantitative analysis of phytosterols in edible oils using APCI liquid chromatography–tandem mass spectrometry. Lipids, 48(9), pp.949-956.

Murphy, K.J., Chronopoulos, A.K., Singh, I., Francis, M.A., Moriarty, H., Pike, M.J., Turner, A.H., Mann, N.J. and Sinclair, A.J., 2003. Dietary flavanols and procyanidin oligomers from cocoa (*Theobroma cacao*) inhibit platelet function. The American Journal of Clinical Nutrition, 77(6), pp.1466-1473.

Nakagawa, H., Takaishi, Y., Tanaka, N., Tsuchiya, K., Shibata, H. and Higuti, T., 2006. Chemical Constituents from the Peels of Citrus Sudachi. Journal of Natural Products, 69(8), pp.1177-1179.

Rao, J.U.M., Giri, G.S., Hanumaiah, T. and Rao, K.V.J., 1986. Sampangine, a new alkaloid from *Cananga odorata*. Journal of Natural Products, 49(2), pp.346-347.

Rasul, A., Millimouno, F.M., Ali Eltayb, W., Ali, M., Li, J. and Li, X., 2013. Pinocembrin: a novel natural compound with versatile pharmacological and biological activities. BioMed Research International, 2013.

Tanaka, T., Orii, Y., Nonaka, G.I., Nishioka, I. and Kouno, I., 1996. Syzyginins A and B, two ellagitannins from *Syzygium aromaticum*. Phytochemistry, 43(6), pp.1345-1348.

Wiehler, G. and Marion, L., 1958. Homostachydrine, a new alkaloid isolated from the seeds of *Medicago sativa* L. Grimm. Canadian Journal of Chemistry, 36(2), pp.339-343.

Yannai, S., Dictionary of food compounds with CD-ROM: Additives, flavors, and ingredients. 2003. Boca Raton: Chapman & Hall/CRC Press.

Zhang, Y., Wang, D., Lee, R.P., Henning, S.M. and Heber, D., 2009. Absence of pomegranate ellagitannins in the majority of commercial pomegranate extracts: implications for standardization and quality control. Journal of Agricultural and Food Chemistry, 57(16), pp.7395-7400.
