# Supplementary material for: Metabolic profiling and scavenging activities of developing circumscissile fruit of psyllium (Plantago ovata Forssk.) reveal variation in primary and secondary metabolites
Source: BMC Plant Biol. 2020 Mar 14;20:116. doi: 10.1186/s12870-020-2318-5 (PMC7071626; doi:10.1186/s12870-020-2318-5)
Supplement: Supplementary file 4 — Additional file 4: Figure S1. Loading plot of PC analysis (PCA) based on (A) fatty acid composition, (B) amino-acids and (C) total flavonoid and phenolic contents of developing psyllium fruit. [file 12870_2020_2318_MOESM4_ESM.pdf]

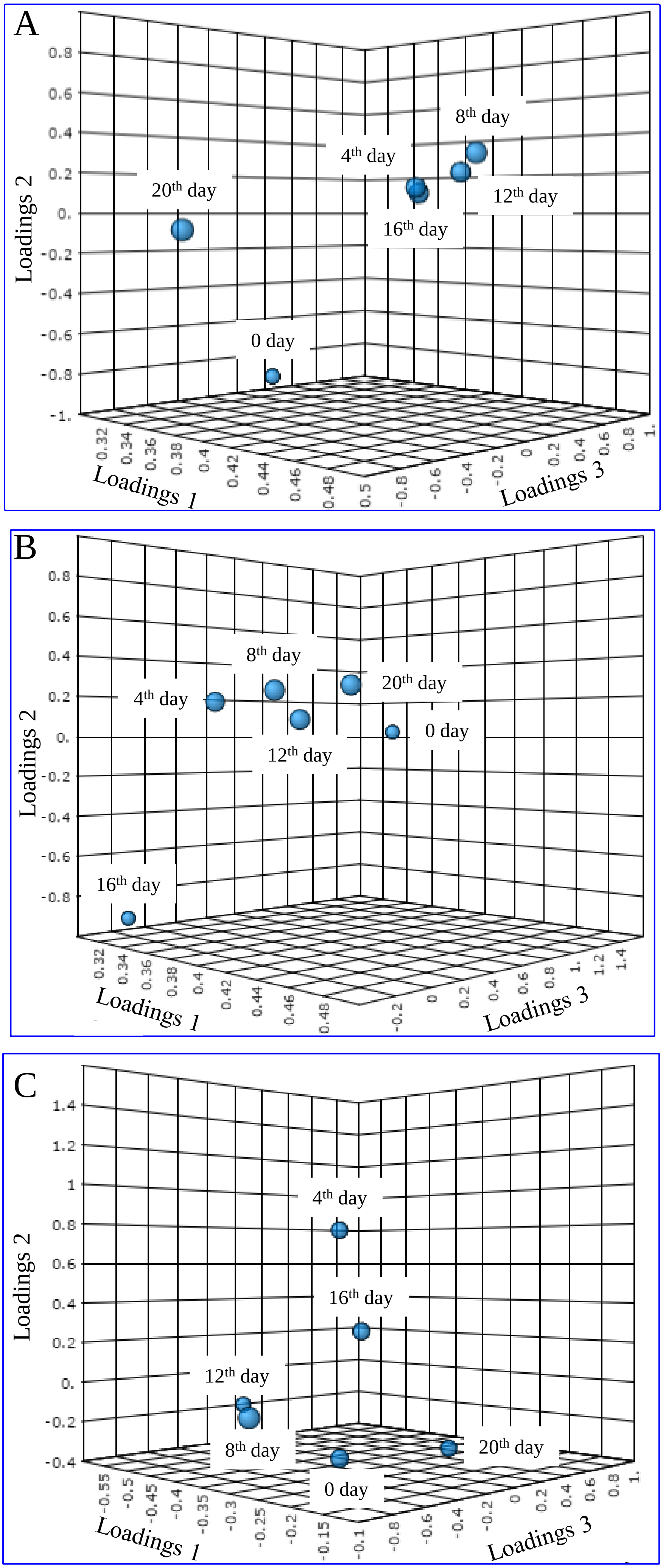

Figure S1: Loading plot of PC analysis (PCA) based on (A) fatty acid composition, (B) amino-acids and (C) total flavonoid and phenolic contents of developing psyllium fruit.
